# Educational initiatives and implementation of electroencephalography into the acute care environment: a protocol of a systematic review

**DOI:** 10.1186/s13643-020-01439-x

**Published:** 2020-08-10

**Authors:** Shaurya Taran, Wael Ahmed, Esther Bui, Lara Prisco, Cecil D. Hahn, Victoria A. McCredie

**Affiliations:** 1 Interdepartmental Division of Critical Care, Li Ka Shing Knowledge Institute, 204 Victoria Street, 4th Floor, Room 411, Toronto, Ontario M5B 1T8 Canada; 2 Department of Critical Care Medicine, Sunnybrook Health Sciences Center, Toronto, Ontario Canada; 3 Division of Neurology, University Health Network, Toronto, Ontario Canada; 4 Neurosciences Intensive Care Unit, John Radcliffe Hospital, Oxford, UK; 5 Nuffield Department of Clinical Neurosciences, University of Oxford, Oxford, UK; 6 Division of Neurology, The Hospital for Sick Children, and Department of Pediatrics, University of Toronto, Toronto, Ontario Canada; 7 Division of Critical Care Medicine, Department of Medicine, University Health Network, Toronto, Ontario Canada; 8 Krembil Research Institute, University Health Network, Toronto, Ontario Canada

**Keywords:** Electroencephalography, EEG, QEEG, Education, Critical care, Instruction, Teaching program

## Abstract

**Background:**

Use of electroencephalography (EEG) is currently recommended by the American Clinical Neurophysiology Society for a wide range of indications, including diagnosis of nonconvulsive status epilepticus and evaluation of unexplained disorders of consciousness. Data interpretation usually occurs by expert personnel (e.g., epileptologists, neurophysiologists), with information relayed to the primary care team. However, data cannot always be read in time-sensitive fashion, leading to potential delays in EEG interpretation and patient management. Multiple training programs have recently been described to enable non-experts to rapidly interpret EEG at the bedside. A comprehensive review of these training programs, including the tools used, outcomes obtained, and potential pitfalls, is currently lacking. Therefore, the optimum training program and implementation strategy remain unknown.

**Methods:**

We will conduct a systematic review of descriptive studies, case series, cohort studies, and randomized controlled trials assessing training programs for EEG interpretation by non-experts. Our primary objective is to comprehensively review educational programs in this domain and report their structure, patterns of implementation, limitations, and trainee feedback. Our secondary objective will be to compare the performance of non-experts for EEG interpretation with a gold standard (e.g., interpretation by a certified electroencephalographers). Studies will be limited to those performed in acute care settings in both adult and pediatric populations (intensive care unit, emergency department, or post-anesthesia care units). Comprehensive search strategies will be developed for MEDLINE, EMBASE, WoS, CINAHL, and CENTRAL to identify studies for review. The gray literature will be scanned for further eligible studies. Two reviewers will independently screen the search results to identify studies for inclusion. A standardized data extraction form will be used to collect important data from each study. If possible, we will attempt to meta-analyze the quantitative data. If heterogeneity between studies is too high, we will present meaningful quantitative comparisons of secondary outcomes as per the synthesis without meta-analysis (SWiM) reporting guidelines.

**Discussion:**

We will aim to summarize the current literature in this domain to understand the structure, patterns, and pitfalls of EEG training programs for non-experts. This review is undertaken with a view to inform future education designs, potentially enabling rapid detection of EEG abnormalities, and timely intervention by the treating physician.

**PROSPERO registration:**

Submitted and undergoing review. Registration ID: CRD42020171208.

## Background

Patients admitted to acute-care units are at high risk for experiencing a variety of neurologic complications [1]. These may arise from primary neurologic pathologies or may be secondary to surgical procedures, trauma, systemic disease processes, or hemodynamic perturbances [2]. Among the most serious neurologic complications are seizures. Clinical suspicion of seizures requires prompt investigation and management. However, a large proportion of acutely ill patients with altered mental status have nonconvulsive seizures that cannot be diagnosed with clinical signs alone [3]. Evidence from animal models and clinical studies suggests a clear association between prolonged seizures and worse neurological outcomes [4, 5], reflecting the importance of early identification and treatment.

Electroencephalography (EEG) is the single most important modality for the diagnosis of seizures. EEG can be recorded continuously at the bedside (continuous EEG, cEEG), has excellent temporal resolution, and is sensitive to rapid changes in neuronal activity [6]. Prolonged cEEG recordings are often expressed as compressed mathematical trends of the raw EEG. The quantitative EEG (QEEG) trends are calculated by specialized software and visualized as plots. A recent consensus statement from the American Clinical Neurophysiology Society (ACNS) recommends the use of cEEG in multiple acute care situations, including in comatose patients after acute brain injury; if impaired consciousness persists following seizures; in cases of unexplained alteration of mental status without known acute brain injury; when neuromuscular blocking drugs are used in high-risk patients; when certain characteristic patterns are identified on routine EEG recording; and to aid prognostication of hypoxic-ischemic encephalopathy after cardiac arrest [7]. Over the past decade, technical advances have improved the efficiency of EEG and cEEG recording, and existing guidelines recommend that cEEG monitoring should be initiated within 1 h of suspicion for non-convulsive seizures [8]. However, EEG and cEEG are not feasible in many hospitals, and even when available there remain important barriers to their use [9, 10]. One such limitation is the lack of 24/7 availability of expert personnel, including electroencephalographers and epileptologists, for prompt EEG placement and interpretation. This has important implications for patient management, including delayed diagnosis and treatment of potentially serious pathologies (e.g., status epilepticus).

Multiple educational interventions have shown that it is possible to enable non-experts to rapidly set up and interpret EEG at the bedside [11–13]. However, a comprehensive overview of these training programs, including the methods used, outcomes obtained, and potential pitfalls, remains lacking. The optimum training program and implementation strategy thus remains unknown. We will therefore conduct a systematic review to outline the structure and patterns of training programs implemented for EEG interpretation by non-experts. As secondary objectives, we will aim to (i) compare the performance of non-experts receiving structured training in EEG interpretation against a gold standard (e.g., EEG interpretation by epileptologists), and (ii) compare trainees’ performance on structured assessments pre- and post-completion of the educational program. We undertake this review to inform future EEG educational designs for non-experts, potentially enabling curriculum developers to select the most impactful, well-received, and high-yield educational interventions. An anticipated benefit of such a curriculum would be to enable non-experts to expediently detect important neurologic pathologies at the bedside.

## Methods

### Design

A team of investigators with expertise in critical care EEG, neurocritical care, adult and pediatric neurology, and health information collaborated to develop the research question and study design. This systematic review will follow the guidelines set out in the Cochrane Handbook for Systematic Review and Meta-Analyses [14]. The protocol has been registered in PROSPERO (ID: CRD42020171208). The research methodology presented in the final manuscript will adhere to the Preferred Reporting Items for Systematic Reviews and Meta-Analyses (PRISMA) guidelines [15]. Deviations from the protocol will be recorded in the final report.

### Information sources and search strategy

MEDLINE, EMBASE, Web of Science (WoS), the Cumulative Index to Nursing and Allied Health Literature (CINAHL), and the Cochrane Central Register of Controlled Trials (CENTRAL) will be systematically searched from their inception date to January 2020. With the collaboration of a health information specialist, the search strategy was carefully developed to capture all studies of potential interest, using a combination of free text keywords and subject headings terms. Keywords used to construct the search included “electroencephalography,” “critical care,” “emergency care,” “curriculum,” and “medical education.” An example of our full search strategy for MEDLINE has been provided as a supplementary file with this protocol.

A review of the gray literature will be performed to identify unpublished or ongoing studies using Google Scholar, https://clinicaltrials.gov, and http://www.controlled-trials.com. This systematic review will also include a hand search of the past 10 years of published abstracts from relevant conference proceedings. Before submission for publication, the search will be rerun through each database to account for newly reported findings. Authors of included studies will be contacted up to 3 times to clarify any unclear or unavailable information as necessary. A step-by-step breakdown of included and excluded studies will be provided in the final manuscript using the standard PRISMA flow diagram.

### Eligibility criteria and study selection

We will apply the following eligibility criteria to identify studies for inclusion in this review: (1) randomized controlled trials, pre-post interventional studies, and observational studies including descriptive and cohort studies (2) examining adult or pediatric patients in whom EEG recording (including intermittent/short EEG, processed EEG (e.g., Bis, entropy, Sedline), video EEG, cEEG, and QEEG) was performed. (3) Implementation of EEG must occur in either the intensive care unit (ICU), emergency department (ED), or post-anesthetic care unit (PACU) setting, (4) with data analysis performed by a non-expert in EEG interpretation (e.g., bedside nurse, intensivist, ED physician, anesthetist, or trainee physician). (5) Studies must include a program of structured training in EEG interpretation. Cluster RCTs will not be included, since the unit of allocation of included studies must be the individual. Studies will be excluded if they (1) do not adequately describe the structure and content of training programs, (2) are applied to subjects with specialized knowledge of EEG (e.g., neurophysiologists, epileptologists), (3) or if they are conducted in a setting other than the ICU, ED, or PACU. Studies involving medical students, residents, and clinical fellows will be eligible for consideration as long as they meet the remainder of the inclusion criteria. Records will be screened and managed by two reviewers independently using Endnote, version 9.0 (Clarivate Analytics).

Two reviewers (ST, WA) will independently screen all material generated by the search algorithm to identify studies for inclusion. Each reviewer will be blinded to the others’ appraisal of the literature. Studies will initially be screened by title, keywords, and abstract to determine article eligibility. Articles passing the initial step will be reviewed by two reviewers in full, with articles selected based on eligibility criteria. As a further step, the reference list of each selected study will be scanned to identify additional studies for inclusion. In cases of ambiguity, authors of studies in question will be contacted for clarification of uncertain information, with their response rates tracked and reported in the final manuscript. No restrictions will be applied to language and foreign papers will be translated to English. Abstracts without a corresponding full-length manuscript will be considered if they present sufficient material on educational design, implementation, and outcomes. In cases of incomplete information, authors of abstracts will be contacted up to 3 times to obtain further details of the study; studies will be excluded in the event of non-response. If there are discrepancies in the final study lists generated by the two reviewers, a third reviewer will be consulted for arbitration. Reasons for exclusion of studies assessed in full will be presented in the final published manuscript.

### Data collection

Two independent reviewers (ST, WA) will extract data from each study in the final list into a standardized pre-piloted data collection form. We will collect and report data on (1) study design, including but not limited to study type, year of publication, inclusion and exclusion criteria, treatment setting (e.g., ICU, ED, or PACU), sources of funding, and conflicts of interest; (2) baseline patient characteristics including age, sex, comorbidities, and primary pathology (neurological (e.g., traumatic brain injury, subarachnoid hemorrhage, epilepsy) vs non-neurological (e.g., sepsis, acute respiratory distress syndrome (ARDS), trauma)); (3) indication for EEG; (4) type of medical or surgical treatment received, including use of mechanical ventilation or tracheostomy; (5) characteristics of the EEG educational program, including mode of instruction (e.g., self-guided, didactic, case-based); (6) duration of the training program, and whether follow-up sessions were organized; (7) methods for assessing trainee performance (e.g., written quiz, bedside interpretation of EEG recordings); (8) outcome of the intervention (e.g., improvement in EEG interpretation capabilities by the trainee); (9) and trainee feedback on the educational intervention (if reported). The initial data extraction form will be piloted on five included studies to ensure robustness, with subsequent modifications for thoroughness performed if necessary. A template of the data extraction form is appended in the supplementary file. Discrepancies in extracted data will be resolved in discussion between the two primary reviewers. Duplicated studies will be included only once in the final analysis, with the most comprehensive article being represented.

### Assessment of methodological quality and risk of bias

If our search identifies a randomized controlled trial (RCT) deemed eligible for inclusion, its risk of bias will be evaluated by two independent reviewers with the Cochrane Collaboration’s risk of bias tool [14]. However, we anticipate the majority of studies to be descriptive studies and pre-post interventional studies. For the assessment of these studies, we will use the National Institute of Health (NIH) quality assessment tools [16]. NIH checklists will be adapted for each study design. Each checklist includes items for evaluating potential flaws in study methods or implementation, including sources of bias (e.g., patient selection, performance, attrition, and detection), confounding, study power, the strength of causality in the association between interventions and outcomes, and other factors. Quality reviewers may select “yes,” “no,” or “cannot determine/not reported/not applicable” in response to each item on the tool. Studies are assigned a quality rating of “good,” “fair,” or “poor” based on the aggregate total of “yes” responses. Summary reports on the quality of each represented study will be presented in tables in the final manuscript. The assessment of both randomized and non-randomized studies will be undertaken independently by two reviewers (ST, WA), with discrepancies resolved after joint article review and discussion.

### Outcomes

Our primary objective is to comprehensively review educational programs for training non-experts to interpret EEG, with a focus on reporting programs’ structure, patterns of implementation, challenges, and trainee experience. Results will be presented as a narrative summary, with information grouped by theme (e.g., program structure, methods of evaluation, trainee experience). As secondary objectives, we will (i) aim to compare the performance of non-experts receiving structured training in EEG interpretation against a gold standard (e.g., EEG interpretation by an epileptologist or neurophysiologist), and (ii) compare trainees’ performance pre- and post-completion of the educational program. At the minimum, the ability of trainees to accurately detect seizures will be compared with experienced interpreters. Additionally, if studies permit, trainees will also be assessed in their ability to recognize other important EEG abnormalities, including (but not limited to) alpha coma, periodic lateral epileptiform discharges (PLEDs), and generalized periodic discharges (GPEDs). If available, performance in the assessment of raw EEG and QEEG by non-expert personnel will be also assessed.

Agreement in interpretation of EEG between non-neurologists and experts will be assessed with the kappa coefficient (*k*), and the strength of agreement will be interpreted with the Landis and Koch classification (< 0 = very bad; 0-0.2 = slight; 0.21-0.4 = fair; 0.41-0.6 = moderate; 0.61-0.8 = good; and 0.81-1 = almost perfect) [17]. For the assessment of both secondary objectives, only studies reporting the relevant data will be included.

### Statistical analysis and data synthesis

Data will be extracted from the standardized form and presented in a descriptive manner. Categorical data will be reported in proportions while continuous data will be presented as means with standard deviations or medians with ranges depending on the format used in the primary studies. We expect considerable heterogeneity in educational design, training duration, and use of performance metrics, potentially prohibiting meta-analysis. The Cochrane Handbook outlines methods to synthesize findings if meta-analysis cannot be performed, for example, due to heterogeneity [14]. In our review, we will follow the Cochrane methods if meta-analysis is deemed inappropriate. In addition, we will adhere to a rigorous reporting methodology as described by the synthesis without meta-analysis (SWiM) guidelines [18]. The SWiM guideline is a 9-item reporting checklist that outlines standardized metrics used for synthesis, the synthesis method, a summary of findings, and limitations of the synthesis. A protocol for the synthesis and analysis of studies is presented using the SWiM format, although modifications may be required based on the final pool of available studies.

1. Grouping studies for synthesis: Studies being analyzed quantitatively will be separated by design into two groups. Pre-post interventional studies assessing non-expert performance before and after an EEG training program will constitute the first group. Studies assessing non-expert performance against a gold standard (e.g., EEG interpretation by an electroencephalographer) will constitute the second group. For individual studies reporting both measures, data and outcomes will be reported separately.
2. Standardized metric used: For the comparison of EEG interpretation by non-experts with experts, the kappa coefficient will be used to standardize assessments. If the kappa statistic is not reported, it will be calculated using data from the corresponding 2 × 2 table. In cases of incomplete data, authors will be contacted to supply the data. For pre-post comparisons, the chosen measure of intervention effect will be the *P* value. If the *P* value is not presented, data from the study will be used to perform the *P* value calculation. In cases of incomplete data, authors of the study will be contacted.
3. Synthesis method: For non-expert vs. expert comparisons, a forest plot of the summary statistic (i.e., kappa coefficient) will be displayed along with the corresponding 95% confidence intervals for each study. Studies will be pooled using random-effects models (DerSimonian and Laird method), and a summary estimate will be calculated as a weighted average of the kappa values from individual studies. Statistical calculations will be performed using Review Manager 5.3. For pre-post studies, we will use random-effects models to calculate pooled estimates of effect sizes using the Review Manager 5.3.5 software (Cochrane Collaboration, Oxford, UK). Pooled continuous-effect measures will be expressed as mean differences (MD) with 95 % confidence intervals (CI). Assuming there are a reasonable number of studies, we will plan to perform a subgroup analysis to examine for heterogeneity of the effect across subgroups. The following subgroups will be examined: clinical background of the participant (e.g., nurse or physician), duration of the training program, and type of EEG reviewed (e.g., aEEG, CDSA, short/intermittent EEG). We will perform a *z* test of interaction for all subgroup comparisons, which tests the null hypothesis that the intervention effects in each subgroup are the same. If there is considerable clinical heterogeneity, we will aim to combine *P* values based on the direction of effect and the precise *P* value of individual studies using Fisher’s method. Results will be displayed visually in the form of an albatross plot, following the recommendation from the Cochrane Handbook [14].
4. Criteria used to prioritize results: No restrictions will be applied to studies being synthesized. However, given that studies are likely to vary in their risk of bias, the final quality of each study (“good,” “fair,” or “poor”) as determined by the NIH quality assessment tool will be displayed alongside the results to contextualize readers’ interpretation of the synthesis.
5. Investigation of heterogeneity: If formal statistical analysis is possible, we will use the *I*^*2*^ statistic to evaluate percentage variance that is attributable to study heterogeneity. Interpretations regarding the significance of heterogeneity will be made as per standard characterization as negligible (< 40%), moderate (30-60%), substantial (50-90%), or considerable (75-100%) [14]. In case formal statistical analysis is not possible, informal methods will be used to investigate heterogeneity in reported effects. Tables and figures will be ordered in the final manuscript based on hypothesized modifiers, including methodologic characteristics (e.g., study design), subpopulations (e.g., clinical background of the participants, number of years in practice, setting where the program was administered (e.g., ICU or ED)), and other contextual factors. Where possible, results from studies will be grouped and presented according to the degree of heterogeneity. This method is in line with the Cochrane Handbook’s recommendation to group studies by characteristics that might enhance their interpretation.
6. Certainty of evidence: Assessment of the certainty of evidence will take place according to the GRADE recommendations. However, assessing some domains is anticipated to be challenging, and if formal assessment cannot be adequately performed, this step will be excluded in the final analysis. For the assessment of publication bias, funnel plots will be constructed and visually inspected for asymmetry.
7. Data presentation methods: Data will be presented in figures and tables in the final manuscript, grouped by the secondary outcome of interest. Forest plots of kappa coefficients will be constructed for studies comparing seizure detection between non-expert vs. expert, as described above. For pre-post studies, data will either be presented as forest plots of the mean test scores or *P* values will be displayed as an albatross plot.
8. Reporting results: For each secondary objective, the synthesized findings will be described, making clear which studies contributed to the findings and the synthesis method used. Findings will not be over-interpreted and will be presented in the context of the quality of studies informing them.
9. Limitations of the synthesis: Limitations will be described, including those resulting from limited evidence, incompletely reported outcome, or incomplete effect estimates.

## Discussion

EEG is an important tool to evaluate neurologic function, and its use is currently recommended by multiple professional societies for a wide variety of indications [7, 19]. However, while uptake in EEG has increased substantially in the past decade [20], an important challenge remains obtaining expert interpretation of data (e.g., by epileptologists) in a timely fashion. As a case in point, a survey of neurophysiologists and neurointensivists across approximately 100 institutions in the USA indicated that only 22% of hospitals had dedicated staff for cEEG 24/7 [21]. Delays in EEG interpretation may contribute to patient morbidity and mortality and increase healthcare costs.

Multiple studies have described training programs for non-experts to rapidly interpret bedside EEG. A comprehensive review of these training programs, including the tools used, outcomes obtained, and potential pitfalls, is currently lacking. Thus, the optimum training program and implementation strategy remains unknown. This is an area worth investigating, since EEG interpretation by bedside personnel may allow for expedient detection of important pathologies while awaiting a formal and complete interpretation by trained experts. In this systematic review, we seek to collect and synthesize data from all studies looking at the use of educational programs to train non-experts in EEG interpretation. Our findings may inform future EEG curriculum design in important ways, most notably by allowing curriculum experts to choose impactful and high-quality educational strategies while avoiding those shown to be less efficacious. Furthermore, it is currently unknown whether EEG educational programs improve non-experts’ knowledge of core principles, and also whether non-experts may achieve acceptable rates of seizure detection compared to experts. Our systematic review will help answer these important questions and potentially bolster interest in the use of EEG educational interventions for non-experts.

The methodology and study design of this systematic review will adhere to well-recognized standards of quality assurance [15]. We will aim to assess the methodologic quality of the included studies and examine their risk of bias. However, our efforts to explore secondary objectives for this topic may be challenging due to the anticipated heterogeneity between studies. Foreseeable limiting factors include differences in educational design, duration, and performance metrics used. Our final assessment will include a global evaluation on the quality of reported studies published in this field. We plan to present the results of this systematic review at research conferences and aim to publish our findings in a peer-reviewed journal.

## Supplementary information

**Additional file 1.** PRISMA-P 2015 Checklist.

**Additional file 2.** Medline Search Strategy.

**Additional file 3.** Data Extraction Form for Eligible Studies.

## Data Availability

The datasets used and analyzed in this review are available from the corresponding author (ST) on reasonable request.

## Abbreviations

EEG: Electroencephalography: The measurement of electrical activity in different parts of the brain and the recording of such activity as a visual trace
cEEG: Continuous Electroencephalography: A neuromonitoring modality that allows for uninterrupted assessment of cerebral electrical activity, with results displayed as a visual trace
QEEG: Quantitative electroencephalography: A procedure that processes the recorded EEG activity from a multi-electrode recording using a computer. This multi-channel EEG data is processed with various algorithms. The digital data is statistically analyzed, and the processed EEG is commonly converted into color maps
ED: Emergency department: The department of a hospital that provides immediate treatment for acute illnesses and trauma
GPED: Generalized periodic epileptiform discharges: Periodic complexes occupying at least 50% of a standard 20-min EEG over both hemispheres in a symmetric, diffuse, and synchronized manner
GRADE: Grading of Recommendations, Assessment, Development, and Evaluations: A working group established in 2000 that aims to develop a common, sensible, and transparent approach to grading quality (or certainty) of evidence and strength of recommendations
ICU: Intensive care unit: A department of the hospital where patients who are severely ill are kept under close observation and provided life-sustaining therapies
PACU: Post-anesthesia care unit: An acute-care unit where patients are typically observed following surgery and administration of anesthesia
PLED: Periodic lateralized epileptiform discharge: An abnormal EEG pattern consisting of unilateral, focal spike or sharp wave complexes with a period appearance usually at a rate of 1-2 s
PRISMA: Preferred Reporting in Systematic reviews and Meta-analyses: A guideline that outlines an evidence-based minimum set of items for reporting in systematic reviews and meta-analyses
RCT: Randomized control trial: A study design that randomly assigns participants into an experimental group or a control group. As the study is conducted, the only expected difference between the control and experimental groups in an RCT is the experimental variable being studied
SWiM: Synthesis without meta-analysis: A guideline to enable clear reporting in reviews of interventions in which alternative methods to meta-analysis of effect estimates are used
