# Supplementary material for: Educational initiatives and implementation of electroencephalography into the acute care environment: a protocol of a systematic review
Source: Syst Rev. 2020 Aug 10;9:175. doi: 10.1186/s13643-020-01439-x (PMC7418425; doi:10.1186/s13643-020-01439-x)
Supplement: Supplementary file 2 — Additional file 2. Medline Search Strategy. [file 13643_2020_1439_MOESM2_ESM.docx]

# Medline Search Strategy

| **#** | **Searches** | **Results** |
| --- | --- | --- |
| 1 | exp electroencephalography/ or exp brain waves/ or alpha rhythm/ or beta rhythm/ or delta rhythm/ or gamma rhythm/ or theta rhythm/ or electrocorticography/ or exp electroencephalography phase synchronization/ or cortical synchronization/ or magnetoencephalography/ | 155887 |
| 2 | electroencephalogra*.mp,jw. | 156747 |
| 3 | electro-encephalogra*.mp. | 806 |
| 4 | diagnostic techniques, neurological/ and (exp Brain/ or exp Brain Diseases/ or exp Brain Injuries/) | 510 |
| 5 | brain wave?.mp. | 4062 |
| 6 | alpha rhythm?.mp. | 4944 |
| 7 | beta rhythm?.mp. | 2038 |
| 8 | delta rhythm?.mp. | 1612 |
| 9 | gamma rhythm?.mp. | 1019 |
| 10 | theta rhythm?.mp. | 5014 |
| 11 | electrocorticogra*.mp. | 3566 |
| 12 | electro-corticogra*.mp. | 30 |
| 13 | (cortical adj1 synchroni*).mp. | 3112 |
| 14 | magnetoencephalogra*.mp. | 8665 |
| 15 | magneto-encephalogra*.mp. | 149 |
| 16 | continuous EEG?.mp. | 784 |
| 17 | aeeg.ti,ab,kf. | 388 |
| 18 | ceeg.ti,ab,kf. | 266 |
| 19 | cvEEG?.ti,ab,kf. | 10 |
| 20 | iEEG?.ti,ab,kf. | 335 |
| 21 | microEEG?.mp. | 4 |
| 22 | micro-EEG?.mp. | 14 |
| 23 | mEEG?.ti,ab,kf. | 14 |
| 24 | quantitative EEG?.mp. | 1230 |
| 25 | QEEG?.ti,ab,kf. | 864 |
| 26 | spot EEG?.mp. | 2 |
| 27 | sEEG?.ti,ab,kf. | 584 |
| 28 | videoelectroencephalogra*.mp. | 20 |
| 29 | video-electroencephalogra*.mp. | 955 |
| 30 | videoEEG?.mp. | 25 |
| 31 | video-EEG?.mp. | 2566 |
| 32 | vEEG?.ti,ab,kf. | 236 |
| 33 | or/1-32 [ Electroencephalography ] | 170920 |
| 34 | exp Intensive Care Units/ | 81164 |
| 35 | exp Critical Care/ | 56212 |
| 36 | Critical illness/ | 27534 |
| 37 | Critical Care Nursing/ | 1909 |
| 38 | critical care.mp. | 64323 |
| 39 | (critical?? adj2 ill*).mp,kw. | 50446 |
| 40 | intensive care?.mp. | 147568 |
| 41 | neurocritical care?.mp. | 1054 |
| 42 | neuro-critical care?.mp. | 43 |
| 43 | neurointensiv*.mp. | 698 |
| 44 | neuro-intensiv*.mp. | 184 |
| 45 | "burn department*".mp. | 48 |
| 46 | "burn unit?".mp. | 3289 |
| 47 | "burn ward?".mp. | 108 |
| 48 | "coronary care department*".mp. | 8 |
| 49 | "coronary care unit?".mp. | 6828 |
| 50 | "coronary care ward?".mp. | 6 |
| 51 | (CICU or CICUs).mp. | 182 |
| 52 | (CVICU or CVICUs).mp. | 49 |
| 53 | (high* adj2 depend* adj2 department*).mp. | 6 |
| 54 | (high* adj2 depend* adj2 unit*).mp. | 578 |
| 55 | (high* adj2 depend* adj2 ward*).mp. | 37 |
| 56 | (ICU or ICUs).mp. | 46307 |
| 57 | (intens* adj2 therap* adj2 department*).mp. | 45 |
| 58 | (intens* adj2 therap* adj2 unit*).mp. | 753 |
| 59 | (intens* adj2 therap* adj2 ward*).mp. | 37 |
| 60 | (medic* adj2 surg* adj2 ICU?).mp. | 1059 |
| 61 | "med* surg* icu?".mp. | 570 |
| 62 | medsurg icu?.mp. | 0 |
| 63 | MSICU.mp. | 9 |
| 64 | (NICU or NICUs).mp. | 8496 |
| 65 | (post-an?esth* adj2 care*).mp. | 915 |
| 66 | (postan?esth* adj2 care*).mp. | 1983 |
| 67 | "recovery department*".mp. | 13 |
| 68 | "recovery unit*".mp. | 354 |
| 69 | "recovery ward*".mp. | 103 |
| 70 | (surg* adj2 med* ICU?).mp. | 79 |
| 71 | exp Respiration, Artificial/ | 74978 |
| 72 | ventilat*.mp. | 165475 |
| 73 | exp Ventilators, Mechanical/ | 8953 |
| 74 | (an?esthe* adj2 recover*).mp. | 7262 |
| 75 | Anesthesia Recovery Period/ | 5115 |
| 76 | "Delayed Emergence from Anesthesia"/ | 117 |
| 77 | *Acute Disease/ | 8253 |
| 78 | *Catastrophic Illness/ | 568 |
| 79 | acute care unit?.mp. | 372 |
| 80 | acute care ward?.mp. | 175 |
| 81 | acute care department?.mp. | 16 |
| 82 | Tertiary Care Centers/ | 12317 |
| 83 | (tertiary adj1 care).mp. | 43514 |
| 84 | exp Emergency Medicine/ | 13354 |
| 85 | Emergencies/ | 39760 |
| 86 | exp Emergency Medical Services/ | 135395 |
| 87 | Emergency Nursing/ | 6987 |
| 88 | exp Emergency Service, Hospital/ | 74908 |
| 89 | Emergency Treatment/ | 10493 |
| 90 | exp Evidence-Based Emergency Medicine/ | 395 |
| 91 | Hospital Rapid Response Team/ | 733 |
| 92 | Pediatric Emergency Medicine/ | 226 |
| 93 | "accident? and emergenc*".mp. | 4303 |
| 94 | emergency center?.mp. | 571 |
| 95 | emergency centre?.mp. | 153 |
| 96 | emergency department?.mp. | 71676 |
| 97 | emergency room?.mp. | 15546 |
| 98 | emergency service?.mp. | 71053 |
| 99 | emergency unit?.mp. | 1823 |
| 100 | emergency visit?.mp. | 1071 |
| 101 | emergency ward?.mp. | 899 |
| 102 | or/34-101 [ Critical Care / Intensive Care / Emergency Department ] | 637190 |
| 103 | 33 and 102 [ Electroencephalography + ICU/CC ] | 4817 |
| 104 | "Facility Regulation And Control"/ | 3174 |
| 105 | "Joint Commission On Accreditation Of Healthcare Organizations"/ | 7449 |
| 106 | Clinical Competence/ | 90819 |
| 107 | Ed.fs. [Education Floating Subheading] | 272723 |
| 108 | Education, Medical, Continuing/ | 24542 |
| 109 | exp Academic Medical Centers/ | 91562 |
| 110 | exp Accreditation/ | 18648 |
| 111 | exp Certification/ | 17973 |
| 112 | exp Competency-Based Education/ | 3860 |
| 113 | exp Consensus Development Conference/ | 11749 |
| 114 | exp Consensus Development Conferences As Topic/ | 2787 |
| 115 | exp Consensus/ | 11956 |
| 116 | exp Credentialing/ | 54028 |
| 117 | exp Curriculum/ | 83134 |
| 118 | exp Education, Medical, Graduate/ | 67694 |
| 119 | exp Education, Medical/ | 161582 |
| 120 | exp Education, Professional/ | 292966 |
| 121 | exp Educational Measurement/ | 144883 |
| 122 | Evaluation Studies/ | 247852 |
| 123 | Evaluation Studies as Topic/ | 121674 |
| 124 | exp Faculty, Medical/ | 12922 |
| 125 | exp Faculty/ | 34601 |
| 126 | Feasibility Studies/ | 65397 |
| 127 | exp Hospitals, Teaching/ | 50409 |
| 128 | exp Inservice Training/ | 28657 |
| 129 | exp Interdisciplinary Studies/ | 944 |
| 130 | exp International Educational Exchange/ | 3458 |
| 131 | exp Licensure/ | 17386 |
| 132 | exp Medicine/ [ Specialties ] | 1099637 |
| 133 | exp Mentors/ | 10533 |
| 134 | exp Models, Educational/ | 9942 |
| 135 | exp Physicians/ | 135789 |
| 136 | exp Preceptorship/ | 4999 |
| 137 | exp Professional Competence/ | 114052 |
| 138 | exp Schools, Medical/ | 25198 |
| 139 | exp Teaching Materials/ | 117468 |
| 140 | exp Teaching/ | 83838 |
| 141 | Hospitals, Teaching/ | 22521 |
| 142 | Hospitals, University/ | 29195 |
| 143 | Inservice Training/ | 20020 |
| 144 | Pilot Projects/ | 118638 |
| 145 | Professional Competence/ | 24004 |
| 146 | Program Development/ | 28424 |
| 147 | Program Evaluation/ | 61429 |
| 148 | Specialty Boards/ | 3627 |
| 149 | exp Video Recording/ | 40138 |
| 150 | st.fs. [Standards] | 701845 |
| 151 | Training Support/ | 6051 |
| 152 | (evaluat* adj2 (study or studies)).mp. | 519204 |
| 153 | (feasibilit* adj2 (study or studies)).mp. | 70323 |
| 154 | (fellow or fellows or fellowship*).mp,kw. | 24125 |
| 155 | (intern or interns or internship?).mp. | 52554 |
| 156 | (resident or residents or residency or residencies).mp. | 161544 |
| 157 | (skill or skilled or skills).mp,kw. | 181559 |
| 158 | (training? or trainee?).mp,kw. | 366386 |
| 159 | (video* adj2 record*).mp. | 42358 |
| 160 | accredit*.mp,kw. | 29996 |
| 161 | academ*.mp,kw. | 156637 |
| 162 | certif*.mp,kw. | 56122 |
| 163 | competen*.mp,kw. | 208604 |
| 164 | (course or courses).mp. | 533839 |
| 165 | credential*.mp,kw. | 6754 |
| 166 | curricul*.mp,kw. | 91523 |
| 167 | curricula?.mp,kw. | 15878 |
| 168 | educat*.mp,kw. | 915067 |
| 169 | governance.mp,kw. | 10234 |
| 170 | instruction.mp,kw. | 34951 |
| 171 | learn*.mp,kw. | 387698 |
| 172 | licenc*.mp,kw. | 2243 |
| 173 | licens*.mp,kw. | 43741 |
| 174 | mentor*.mp,kw. | 18017 |
| 175 | non-neuro*.mp. | 9074 |
| 176 | nonneuro*.mp. | 3094 |
| 177 | non-specialist??.mp. | 1522 |
| 178 | nonspecialist??.mp. | 610 |
| 179 | non-technologist?.mp. | 0 |
| 180 | nontechnologist?.mp. | 4 |
| 181 | outline?.mp,kw. | 96109 |
| 182 | postgrad*.mp,kw. | 14639 |
| 183 | post-grad*.mp,kw. | 2409 |
| 184 | preceptor*.mp,kw. | 6452 |
| 185 | proctor*.mp,kw. | 710 |
| 186 | (program? or programme?).mp,kw. | 811798 |
| 187 | qualif*.mp,kw. | 41447 |
| 188 | seminar?.mp,kw. | 7983 |
| 189 | standard?.mp,kw. | 1456337 |
| 190 | syllab*.mp,kw. | 6990 |
| 191 | train*.mp,kw. | 459917 |
| 192 | tutor*.mp. | 8547 |
| 193 | videorecord*.mp. | 428 |
| 194 | video-record*.mp. | 29051 |
| 195 | workshop*.mp,kw. | 32089 |
| 196 | or/104-195 [ Education & related terms ] | 5504883 |
| 197 | 103 and 196 [ Eelectroencephalography + ICU/CC + Education ] | 1455 |
| 198 | exp animals/ not (exp animals/ and exp humans/) | 4663383 |
| 199 | 197 not 198 | 1383 |
| 200 | limit 197 to humans | 1375 |
| 201 | 199 or 200 | 1383 |
| 202 | remove duplicates from 201 | 1378 |
