# Supplementary material for: Educational initiatives and implementation of electroencephalography into the acute care environment: a protocol of a systematic review
Source: Syst Rev. 2020 Aug 10;9:175. doi: 10.1186/s13643-020-01439-x (PMC7418425; doi:10.1186/s13643-020-01439-x)
Supplement: Supplementary file 3 — Additional file 3. Data Extraction Form for Eligible Studies. [file 13643_2020_1439_MOESM3_ESM.docx]

**Data Extraction Form for Eligible Studies**

Reviewer: _____________________ Date of Extraction: ___________________

(dd/mm/yyyy)

**Article Identification:**

RefWorks ID#: _______________

Title: _____________________________________________________________________________________________________________________________________________________________________________________________________________________________________________________________________

First Author: _______________________________

Senior Author: _______________________________

Journal Name/Reference Source: ________________________

Publication type:

- Journal Article
- Published Abstract
- Conference Proceeding
- Other: ___________

Year: _______ Volume: _______ Starting Page: _______

Full Publication: ⁫ Yes ⁫ No Comment: ____________________________

Language of Publication: English Other: _________________________________

Source of sponsorship (Study): ________________________________________________________

Study – Country of Origin: __________________________________________________

Number of Centers Participating: __________________________________________________

Authors contacted for additional data ⁫ Yes ⁫ No

**Verification of Eligibility:**

**Inclusion Criteria:** (verified by “tick” mark)

- Case series, descriptive studies, cohort study OR Randomized Control Trial
- Adult OR pediatric patients received EEG (either intermittent/short EEG, video EEG, continuous EEG (cEEG), processed EEG (e.g., BIS, entropy) or quantitative EEG (QEEG)) for a pre-specified indication (e.g. seizure diagnosis)
- Study was conducted in one of the following inpatient settings: emergency department (ED), intensive care unit (ICU), or post anesthetic care unit (PACU)
- Study described training/educational programs for interpretation of EEG (either raw OR processed data)
- Participants did not have prior expertise in EEG interpretation
- Study reported sufficient details/data of the training program, including but not limited to, structure, content covered, duration, assessment methods, and trainee feedback

*ONLY for assessment of secondary outcomes, include the following:*

- Studies provided sufficient data to calculate performance pre- and post- training program, AND/OR inter-rater agreement coefficient with experienced cEEG readers

**Study Design:**

**Type of Study:**

- Randomized controlled trial
- Retrospective cohort study
- Prospective cohort study
- Pre-post interventional study
- Cross-sectional study
- Case series
- Descriptive study

**Inclusion Criteria:**

- - - 1. ___________________________________________________________________________
      2. ___________________________________________________________________________
      3. ___________________________________________________________________________
      4. ___________________________________________________________________________
      5. ___________________________________________________________________________

**Exclusion Criteria:**

1. ___________________________________________________________________________
2. ___________________________________________________________________________
3. ___________________________________________________________________________
4. ___________________________________________________________________________
5. ___________________________________________________________________________

**Screening and Enrollment:**

For RCTs and Cohort studies

Total participants enrolled: _____ Control pts: ________ Intervention pts: ___________

Timing of enrolment (mm/yy): Control pts: ________ Intervention pts: ____________

Consecutive participants: ⁫ Yes ⁫ No ⁫ Not specified

Description of why not: ____________________________________________________________

Participant identification:

- ICU database
- Prospective enrolment
- Other: __________________
- Hospital database
- Medical records coding

For all other studies

Total participants enrolled: ________

Timing of enrolment (mm/yy):

Consecutive participants: ⁫ Yes ⁫ No ⁫ Not specified

Description of why not: ____________________________________________________________

**Study setting:**

- ED
- ICU (describe specific setting, e.g. MS-ICU, TN-ICU): ______________________________
- PACU
- Mixed setting: _______________________________________________________________

**Study Participant Characteristics:**

Non-expert review by:

- Intensive care physicians
- Neurologists (NOT epileptologists/neurophysiologists)
- ED physicians
- Anesthesiologists
- Nurses
- Other: _______________________
- Mixed group: __________________

Median years in practice (if reported): ____________

Involvement of trainees:

- Yes (describe level and background): __________________________________________
- No
- Not specified

Number of non-expert participants: ______

Expert review by:

- Neurophysiology technologist/ Electroencephalographer
- Neurophysiology doctors
- Neurology nurses
- Neurologist with specialization in epileptology
- Other: _______________________
- Mixed group: __________________

Number of experts performing gold-standard interpretations: ______

**Patient Characteristics:**

- Adult (age > 18 yrs)
- Pediatric (age < 18 yrs)
- Neonate (age < 4 wks)
- Mixed group: _______________________________________________________________

**Clinical diagnosis on admission:**

Neurologic

- - Seizure
  - Traumatic brain injury
  - Subarachnoid hemorrhage
  - Stroke
    1. Hypertensive
    2. Ischemic
  - Hypoxic ischemic encephalopathy (e.g. post cardiac arrest)
  - Other: ________________________

Non-neurologic

- Sepsis
- Acute respiratory distress syndrome (ARDS)
- Trauma
- Other: ________________________

**Aspects of EEG recordings:**

- Short/intermittent EEG (raw)
- Continuous EEG (cEEG) (i.e., >4 hours recordings)
- Quantitative EEG (QEEG)
- Simplified processed EEG (Sedline, Bis, Entropy, CSA, etc)
- Other: _________________________

**Clinical indications for EEG recording:**

- - Suspected non-convulsive status epileptics (NCSE)
  - Convulsive status epilepticus
  - Monitoring treatment response for seizures
  - Monitoring depth of sedation
  - Abnormal movements of unclear etiology
  - Altered mental status NYD
  - Toxic/metabolic encephalopathy
  - Not reported

Total hours of cEEG recorded: __________

Total number of cEEGs obtained: ________

**EEG setup:**

- Full montage (i.e., 21 electrode)
- Simplified full-scalp montage (i.e., 8-16 electrodes)
- Limited montage (e.g., 2 or 4 electrodes limited to one scalp region)
- Not reported

**Training program characteristics:**

Did program involve the following:

- PowerPoint
- Self-study
- Simulations
- Bedside teaching

Description of training program (describe specific methods, duration, content covered, and any other relevant aspects:

________________________________________________________________________________

________________________________________________________________________________

________________________________________________________________________________

________________________________________________________________________________

________________________________________________________________________________

Description of assessment strategy (e.g., written quiz, pass/fail, score-based):

________________________________________________________________________________

________________________________________________________________________________

________________________________________________________________________________

Passing mark (if score-based): ­­­­­­____________

Retention strategy (if described): ________________________________________________________________________________________________________________________________________________________________

Trainee feedback (if reported): ________________________________________________________

_______________________________________________________________________________

Cost of program: ________________________________________________________________

**Patient Baseline Characteristics*:**

|  | **Control (n = )** | **Intervention (n = )** |
| --- | --- | --- |
| Demographics | | |
| Age (SD) |  |  |
| Male Sex |  |  |
| APACHE (SD) |  |  |
| SOFA/MODS/SAPS |  |  |
| Admission GCS (SD) |  |  |
| Post-op admission (Y/N) |  |  |
| Transferred from another institution (Y/N) |  |  |
| Admission data | | |
| Patients with status epilepticus (%) |  |  |
| Patients with pre-existing epilepsy (%) |  |  |
| Post-cardiac arrest (%) |  |  |
| Primary diagnosis was stroke (%) |  |  |
| Primary diagnosis was TBI (%% |  |  |
| Primary diagnosis was SAH (%) |  |  |
| Primary diagnosis was non-neurologic (e.g., sepsis, ARDS, %) |  |  |
| Baseline Hemodynamics | | |
| BP |  |  |
| HR |  |  |
| T_max_ |  |  |
| GCS |  |  |
| SpO2 |  |  |

*For case series, omit “control” arm. Leave fields unfilled if data was not reported.

**Primary Outcome:**

Provide qualitative summary and description of educational program: _____________________

_____________________________________________________________________________

_____________________________________________________________________________

_____________________________________________________________________________

**Secondary Outcomes**

For pre-post-studies*:

| **Variable** | **Pre-program** | **Post-program** |
| --- | --- | --- |
| Overall score | | |
| % answers correct |  |  |
| Sensitivity |  |  |
| Specificity |  |  |
| PPV |  |  |
| NPV |  |  |
| p-value |  | |
| Background activity | | |
| % answers correct |  |  |
| Sensitivity |  |  |
| Specificity |  |  |
| PPV |  |  |
| NPV |  |  |
| p-value |  | |
| Artefact | | |
| % answers correct |  |  |
| Sensitivity |  |  |
| Specificity |  |  |
| PPV |  |  |
| NPV |  |  |
| p-value |  | |
| Sleep-wake cycle | | |
| % answers correct |  |  |
| Sensitivity |  |  |
| Specificity |  |  |
| PPV |  |  |
| NPV |  |  |
| p-value |  | |
| Pathologic trace (specify which type if available) | | |
| % answers correct |  |  |
| Sensitivity |  |  |
| Specificity |  |  |
| PPV |  |  |
| NPV |  |  |
| p-value |  | |
| Burst suppression | | |
| % answers correct |  |  |
| Sensitivity |  |  |
| Specificity |  |  |
| PPV |  |  |
| NPV |  |  |
| p-value |  | |
| Seizure detection | | |
| % answers correct |  |  |
| Sensitivity |  |  |
| Specificity |  |  |
| PPV |  |  |
| NPV |  |  |
| p-value |  | |

*Fill table if study assessed performance of participants on assessments before and after completion of the training program.

For interpretation against gold standard*

| **Variable** | **Trainee** |
| --- | --- |
| Background activity | |
| % answers correct |  |
| Sensitivity |  |
| Specificity |  |
| PPV |  |
| NPV |  |
| Inter-rater agreement coefficient |  |
| Strength of agreement |  |
| Artefact | |
| % answers correct |  |
| Sensitivity |  |
| Specificity |  |
| PPV |  |
| NPV |  |
| Inter-rater agreement coefficient |  |
| Strength of agreement |  |
| Sleep-wake cycle | |
| % answers correct |  |
| Sensitivity |  |
| Specificity |  |
| PPV |  |
| NPV |  |
| Inter-rater agreement coefficient |  |
| Strength of agreement |  |
| Pathologic trace (specify which type if available) | |
| % answers correct |  |
| Sensitivity |  |
| Specificity |  |
| PPV |  |
| NPV |  |
| Inter-rater agreement coefficient |  |
| Strength of agreement |  |
| Burst suppression | |
| % answers correct |  |
| Sensitivity |  |
| Specificity |  |
| PPV |  |
| NPV |  |
| Inter-rater agreement coefficient |  |
| Strength of agreement |  |
| Seizure detection | |
| % answers correct |  |
| Sensitivity |  |
| Specificity |  |
| PPV |  |
| NPV |  |
| Inter-rater agreement coefficient |  |
| Strength of agreement |  |

*Fill table if study assessed performance of participants compared to a “gold-standard” reader (e.g. neurophysiologist/epileptologist
